# Large joints are progressively involved in rheumatoid arthritis irrespective of rheumatoid factor status—results from the early rheumatoid arthritis study

**DOI:** 10.1007/s00296-021-04931-2

**Published:** 2021-08-16

**Authors:** Sizheng Steven Zhao, Elena Nikiphorou, Adam Young, Patrick D. W. Kiely

**Affiliations:** 1 Institute of Life Course and Medical Sciences, University of Liverpool, Liverpool, UK; 2 Centre for Rheumatic Diseases, King’s College London, London, UK; 3 Rheumatology Department, King’s College Hospital, London, UK; 4 Center for Health Services and Clinical Research and Post Graduate Medicine, University of Hertfordshire, Hatfield, UK; 5 Department of Rheumatology, St Georges University Hospitals NHS Foundation Trust, Blackshaw Road, London, UK; 6 Institute for Medical and Biomedical Education, St George’s University of London, London, UK

**Keywords:** Large joints, Ankle, Wrist, Rheumatoid factor, Range of movement, Surgery

## Abstract

This study aimed to examine the progression of large joint involvement from early to established RA in terms of range of movement (ROM) and time to joint surgery, according to the presence of rheumatoid factor (RF). We used a historical longitudinal cohort of early RA patients. Patients were deemed RF negative if all repeated assessments were negative. The rate of progression from normal to any loss of range of movement (ROM) from years 3 to 14 were modelled using generalized estimating equations, for elbows, wrists, hips, knees and ankle, adjusting for confounders. Time to joint surgery was analysed using multivariable Cox models. A total of 1458 patients were included (66% female, mean age 55 years) and 74% were RF-positive. The prevalence of any loss of ROM, from year 3 through to 14 was highest in the wrist followed by ankle, knee, elbow and hip. Odds of loss of ROM increased over time in all joint regions assessed, at around 7–13% per year from year 3 to 14. Time to surgery was similar according to RF-status for the wrist and ankle, but RF-positive cases had a lower hazard of surgery at the elbow (HR 0.37, 0.15–0.90), hip (HR 0.69, 0.48–0.99) and after 10 years at the knee (HR 0.41, 0.25–0.68). Large joints become progressively involved in RA, most frequently affecting the wrist followed by ankle, which is overlooked in composite disease activity indices. RF-negative and positive cases progressed similarly. Treat-to-target approaches should be followed irrespective of RF status.

**Supplementary Information:**

The online version contains supplementary material available at 10.1007/s00296-021-04931-2.

## Introduction

Rheumatoid arthritis (RA) is classically described as a symmetric small joint polyarthritis with additional involvement of large joints [1]. Small joint involvement dominates the classification criteria [2] and scoring tools for disease activity such as the 28-joint disease activity score (DAS28) and the clinical disease activity index (CDAI), where only 8 out of 28 included joints are large [3]. Analysis of joint distribution in RA reveals distinct stable clusters with some phenotypes restricted to small joints, and others involving wrist and large joints [3]. Involvement of large joints is associated with joint destruction and more severe disease [1, 3, 4], with consequences on long-term physical function and quality of life.

The distribution and prevalence of large joint disease assessed by clinical criteria at fixed time points has been provided by data from inception cohorts of early RA [1, 5, 6] and cross-sectional studies in established disease [3, 7]. There is a paucity of information concerning the onset and time course of damage in large joints, such as shoulder, elbow, hip, knee and ankle, from early to established RA, or of the influence of rheumatoid factor (RF) status.

There is a historic perception, now contested, that seronegative patients, who do not have RF and anti-citrullinated peptide (ACPA) antibodies, follow a milder less destructive course than those with these antibodies [8, 9]. This is in part derived from the finding that positive RF is one of the best predictors of small joint erosions [4, 10]. This perception might promote a bias in seronegative patients to select less aggressive treatment, such as non-methotrexate (MTX) conventional synthetic disease modifying anti-rheumatic drug therapy (csDMARD), monotherapy and less rigid adherence to treat to target (T2T) strategies. Evidence to justify this comes from the CARDERA trial, where outcomes in ACPA negative patients were the same in those treated with csDMARD monotherapy as in those receiving intensive combination csDMARD therapy and prednisolone [11].

The historic nature of the Ealy Rheumatoid Arthritis Study (ERAS) provides a unique opportunity to study RA in the context of less aggressive, non-T2T strategies, as was the norm at the time. We aimed to assess individual upper and lower limb large joint progression, specifically in terms of range of movement (ROM) and time to joint surgery, in all patients and according to RF status.

## Methods

### Study population

ERAS was a multi-centre inception cohort of newly diagnosed RA patients (< 2 years disease duration, csDMARD naive), which recruited from nine district general hospitals in England from 1985 to 2001 with yearly follow-up for up to 25 (median 10) years. Patient recruitment into ERAS was based on clinician diagnosis with 70% of patients fulfilling the minimum 1987 American Rheumatism Association criteria [12] at baseline and 96% by last visit. Patients subsequently reclassified as non-RA were excluded from the study. Ethical approval was obtained from East Hertfordshire local research ethics committee and all participants provided written informed consent.

### Treatment profiles

Patients were treated according to contemporaneous practice in each of the ERAS centres, without specific protocols, strategies or other external influences. All centres followed the 1992 good practice guidance [13]. First line treatment was csDMARD monotherapy with/without steroids in > 90%, favouring sulphasalazine (SSZ) from 1986 to 2001, with a gradual switch to MTX monotherapy such that SSZ and MTX were used in equal proportions as first csDMARD by the end of the recruitment period [14]. Combination csDMARDS were generally only used for more severe RA and only a small proportion of patients received bDMARDs [14]. Median time from RA symptom onset to first csDMARD initiation was 8 months.

### Clinical, laboratory, radiographic and surgical outcome measures

Information on demographic, clinical, treatment, laboratory and functional features was recorded at baseline, between 3 and 6 months, at 12 months and then annually on standardized case report forms, as previously described [14, 15]. Disease activity score (DAS) was calculated according to the original three variable method [16].

Range of movement (ROM) of individual shoulder, elbow, wrist, hip, knee, ankle and hindfoot joints was collected at 3, 5, 9 and 12–15 years, the latter assigned as 14 years. ROM was not assessed at baseline (year 0) in this study. Loss of ROM was categorised as normal, < 25%, 26–50%, 51–75%, > 75% reduction and 100% (ankylosis). Any loss of normal ROM was taken to indicate RA involvement in the joint, and involvement of either left or right side was sufficient for that joint region to be designated as involved.

RF was measured in each recruiting centre by routine local laboratory technique, repeated at annual visits. ACPA was not available during the years of recruitment to ERAS. Patients were classified as RF-negative if all assessments for RF were negative, or as RF-positive if any RF result was at least weakly positive.

Radiographs of hands and feet were taken at years 0, 1, 2, 3, 5, 7, 9 and scored according to the Larsen method [17]; scoring wrists, MCPs, PIPs and MTPs for ‘damage’ based on non-erosive joint space narrowing (range 0–50) and ‘erosions’ (0, 1, 2). Radiographs of hands and feet were also assessed for osteoarthritis (OA) according to the Lawrence method [18]. Radiographs were not routinely taken of other large joints.

Surgical procedures at the shoulder, elbow, wrist, knee and ankles were obtained by linking to Hospital Episodes Statistics and the National Joint Registry as previously described [15]. Any operative intervention was included, such as arthroplasty, fusion and arthroscopic surgery.

### Statistics

Descriptive statistics were used to compare patient and disease characteristics by RF status. The rate of progression from normal to any loss of ROM over time (years) was modelled using generalized estimating equations (GEE) with logit link. Models were adjusted for baseline RF-status, age, gender, BMI; and the following time varying covariates: haemoglobin (g/dL), DAS, Health assessment Questionnaire (HAQ), erosions, and osteoarthritis (on radiographs of the hands or feet), each assessed at the same time point as ROM. Missing time-varying covariates were imputed using the last value carried forward. The odds ratios from this model indicate the annual percentage change in odds of losing normal ROM (i.e., transition from normal to any degree of ROM loss) from 3 to 14 years. An interaction term between RF-status and time was used to test the difference in annual change between RF-positive and negative groups. To facilitate interpretation, models were stratified by RF status to provide estimates for each group. Change in the Larsen wrist damage score was modelled using GEE as a continuous variable, while the erosion score was dichotomised into present/absent, using the same covariates as above.

Time to surgery in the RF-positive and negative groups were compared using the Kaplan–Meier estimator and log-rank test. Those with no follow-up time beyond baseline were assigned an arbitrarily small amount of time that did not change the overall patient-time. Cox models were used to derive hazard ratios (HR) adjusted for baseline age, gender and smoking status. Variables violating the proportional hazards assumption (tested using Schoenfeld residuals) were stratified in the model (i.e., allowing strata specific baseline hazards). Where survival curves clearly cross, models were performed separately for the time before and after crossing. Censoring was defined by the last follow-up.

As sensitivity analysis, we additionally adjusted for deprivation (index of multiple deprivation, IMD, 1 indicating most deprived and 5 least), smoking status (categorised as ever, never and missing), and the rheumatic disease comorbidity index (RDCI) [19].

## Results

### Baseline characteristics

A total of 1465 patients were enrolled of whom 1458 (> 99%) had RF data available, constituting the analysis population. RF was measured at baseline in all patients and repeated annually in 62–69% of cases from years 1 to 5 and in 37–56% of cases at each time point from years 6 to 10. Table 1 shows the baseline characteristics. 74% of patients were RF positive. RF-negative patients resided in more deprived areas, had a higher swollen joint count and HAQ, lower ESR and Hb and fewer were ever smokers or had erosions (anywhere in hands and feet). There was no statistical difference between RF-positive and negative patients in gender (66 vs 68% females), baseline age (55 vs 57 years), BMI, DAS, pain VAS, co-morbidities (RDCI), Larsen wrist damage and erosion scores, and Lawrence OA status.

**Table 1:** Baseline characteristics of patients included for analysis

	All patients	RF-negative	RF-positive^a^	*p* value
No. of participants	1465	377	1081	
Age at baseline visit, mean (SD)	55.4 (14.6)	56.6 (15.4)	55.0 (14.3)	0.065
Female	973 (66%)	255 (68%)	715 (66%)	0.60
Ever smoker	388 (42%) (*n* = 915)	72 (35%) (*n* = 204)	316 (44%) (*n* = 711)	0.020
IMD				
1, most deprived	208 (15%)	85 (24%)	123 (12%)	< 0.001
2	228 (17%)	73 (20%)	155 (15%)	
3	278 (20%)	58 (16%)	220 (22%)	
4	280 (20%)	71 (20%)	209 (21%)	
5, least deprived	381 (28%)	74 (20%)	307 (30%)	
BMI, mean (SD)	25.6 (4.5) (*n* = 1267)	26.0 (4.7) (*n* = 310)	25.4 (4.4) (*n* = 957)	0.074
HAQ, mean (SD)	1.1 (0.8) (*n* = 1453)	1.3 (0.8) (*n* = 375)	1.1 (0.8) (*n* = 1078)	< 0.001
DAS, mean (SD)	4.8 (1.3) (*n* = 1446)	4.8 (1.2) (*n* = 375)	4.7 (1.3) (*n* = 1071)	0.61
ESR, median (IQR)	37.0 (18.0, 62.0) (*n* = 1451)	35.0 (16.0, 57.0) (*n* = 375)	38.0 (19.0, 64.0) (*n* = 1076)	0.036
Hb, mean (SD)	12.6 (1.6) (*n* = 1453)	12.4 (1.5) (*n* = 376)	12.7 (1.6) (*n* = 1077)	0.003
Pain VAS, mean (SD)	44.0 (26.4) (*n* = 1405)	43.5 (26.9) (*n* = 356)	44.1 (26.2) (*n* = 1049)	0.71
Swollen joint count, ‘44’ version, median (IQR)	15.0 (7.0, 26.0) (*n* = 1455)	18.0 (9.0, 28.0) (*n* = 377)	14.0 (7.0, 25.0) (*n* = 1078)	< 0.001
RDCI, mean (SD)	0.3 (0.6) (n = 1458)	0.3 (0.6) (*n* = 377)	0.3 (0.6) (*n* = 1081)	0.51
Baseline erosions	1084 (75%)	215 (57%)	869 (81%)	< 0.001
Larsen Wrist damage, mean (SD)	1.3 (5.0) (*n* = 1159)	1.0 (3.7) (*n* = 278)	1.4 (5.3) (*n* = 881)	0.15
Wrist erosion (Larsen score > 0)	70 (6%)	14 (5%)	56 (6%)	0.58
Hands OA^b^	152 (11%)	34 (10%)	118 (12%)	0.37
Feet OA^b^	204 (16%)	56 (18%)	148 (15%)	0.30
Hands joint space narrowing^b^	60 (4%)	14 (4%)	46 (5%)	0.71
Feet joint space narrowing^b^	37 (3%)	6 (2%)	31 (3%)	0.23

*DAS* disease activity score, *Hb* haemoglobin, *HAQ* health assessment questionnaire, *IMD* index of multiple deprivation; *RDCI* rheumatic disease comorbidity index, *VAS* visual analogue scale; (n= participants with available data)

^a^Patients were classified as RF-negative if all assessments were negative, or as RF-positive if any RF result was at least weakly positive

^b^By Lawrence score

### Range of movement in individual joints

**Fig. 1:**
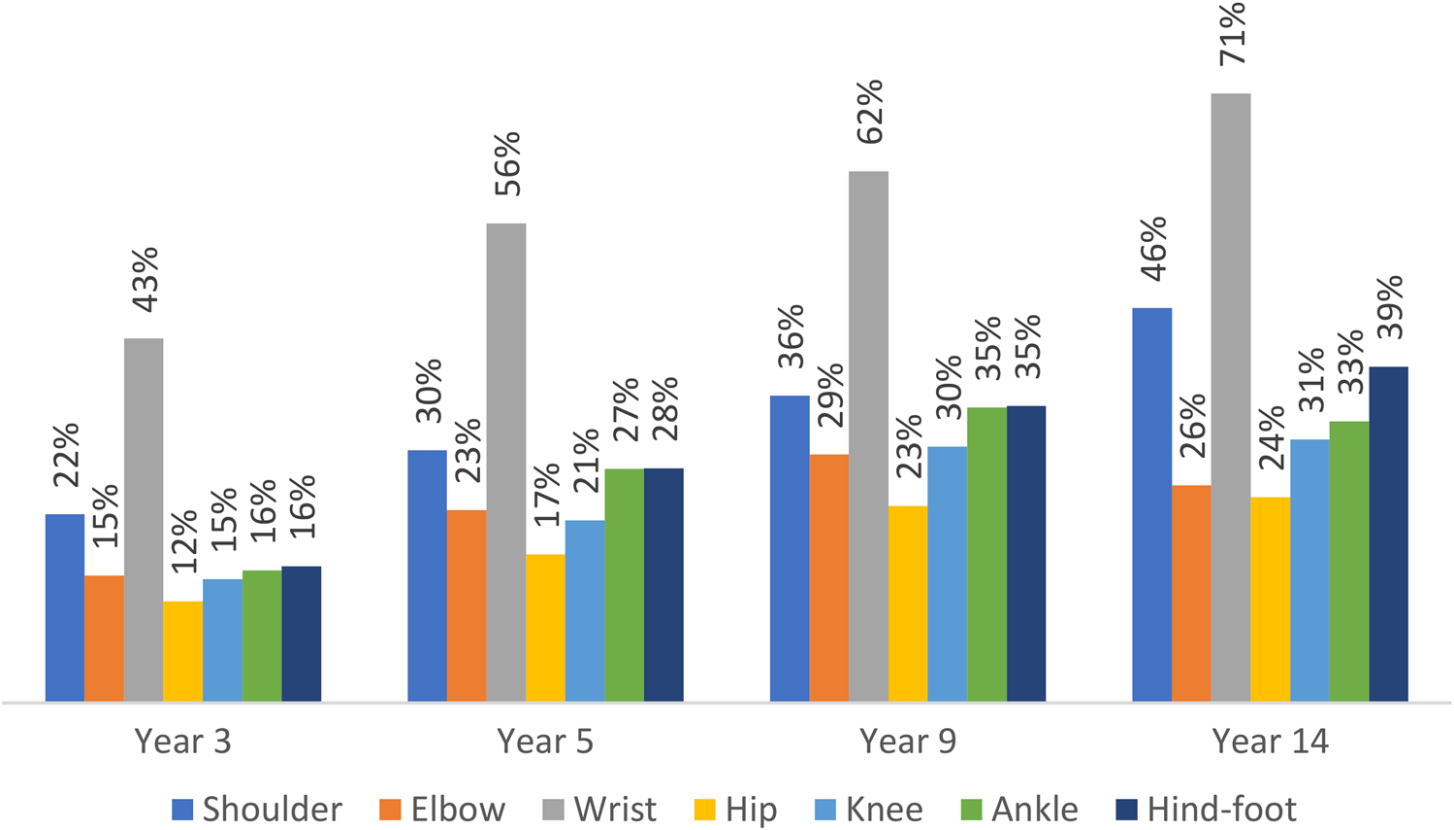
Proportion of participants with any loss of range of movement at each joint over the study period

The proportion of patients with any loss of ROM in either of shoulder, elbow, wrist, hip, knee, ankle and hind foot, measured at year 3, 5, 9 and 14, are shown in Fig. 1 and Supplementary Table S1. The wrist was the joint with the highest proportion of patients exhibiting any loss of ROM, affecting 43% at year 3 and rising sequentially to 71% at year 14. The proportion of patients with any loss of ROM of the elbow, hip, knee and ankle was less than half of those with wrist involvement at all time points, but also incrementally rising through to year 14. Supplementary Figure S1 shows the proportion of patients with differing degrees of loss of ROM per joint at each time point. The proportion of patients at year 9 with greater than 25% loss of ROM was as follows: wrist 30%, ankle 12%, elbow 7%, knee 7% and hip 5%.

**Fig. 2:**
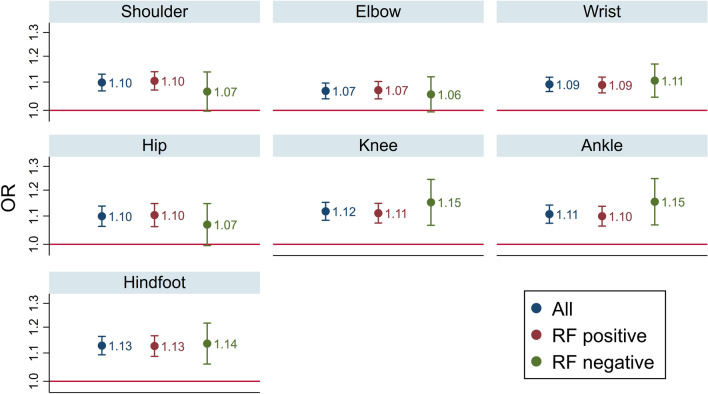
Odds of progression to any loss of ROM (from no loss of ROM) per year in the overall population and stratified by RF status. Models assumed linear progression from 3 to 14 years, adjusted for RF status (in the overall population only), age, gender, BMI, baseline and time-varying erosions, Hb, HAQ, DAS, OA hand and feet joint space narrowing. For example, odds of progression from no to any loss of ROM in the shoulder increased by 10% per year, and did not differ significantly between RF-groups

At the first ROM assessment (year 3), RF-positive participants had significantly lower odds for any loss of ROM at the hip (OR 0.56; 95 CI 0.40, 0.77) and ankle (OR 0.58; 95% CI 0.43, 0.79) compared to RF-negative, whereas there was no significant difference for other joints (online Supplementary Table S1). Modelling showed a statistically significant increase in odds of loss of ROM over time in all joint regions assessed, at around 7–13% per year from year 3 to 14 (Fig. 2). Interaction terms and models stratified by RF-status showed no significant difference between RF-positive and RF-negative patients (Fig. 2). Detailed model coefficients are shown in Supplementary Tables S2-4. Sensitivity analyses additionally adjusting for smoking, RDCI and index of multiple deprivation (IMD) showed no meaningful differences (Supplementary Figure S2).

### Radiographic assessment of the wrist

**Fig. 3:**
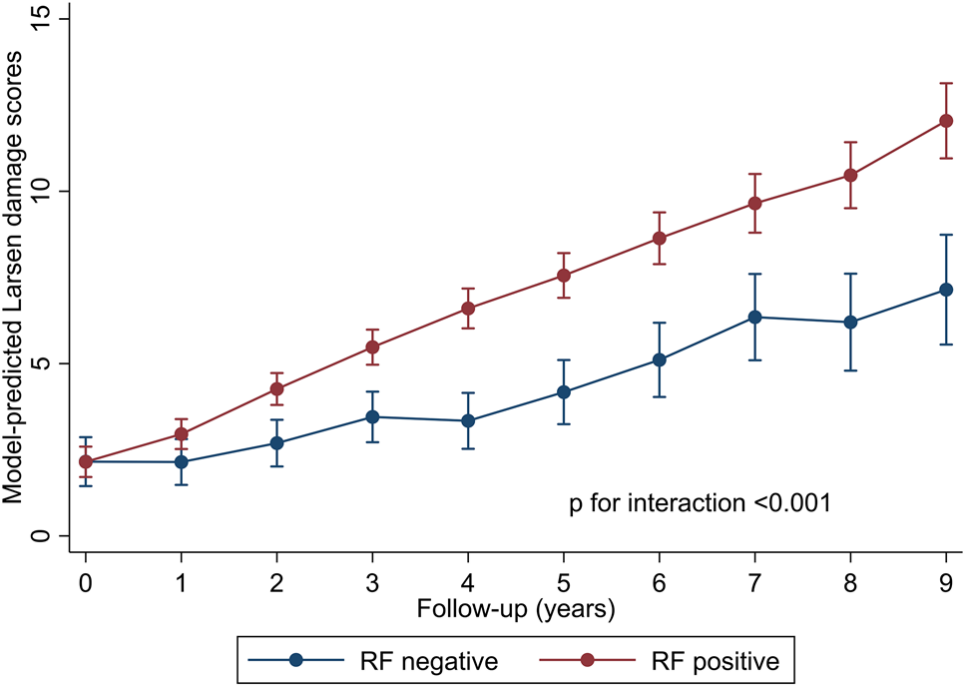
Larsen wrist damage score progression over time according to rheumatoid factor status

RF-positive patients had significantly higher odds of having any erosions at the wrist at year 0 (OR 1.58; 95% CI 1.11, 2.25) versus RF-negatives. Over time (year 0–9), erosions developed (change in Larsen erosion score from 0 to ≥ 1) at the wrist in the entire population, with annual increase OR 1.26 (95% CI 1.22, 1.30). This rate of increase was statistically higher in RF-positives than RF-negatives (*p* for interaction term = 0.013); stratified annual odds of developing any erosions for RF-positive participants was OR 1.28 (95% CI 1.24, 1.32) and for RF-negatives OR 1.17 (95% CI 1.09, 1.26) (full model coefficients in Supplementary Table S5). Larsen damage scores also progressed in all patients (Fig. 3), with linear mixed models revealing a faster rate of progression in the RF-positive patients (full model coefficients in Supplementary Table S6).

### Joint replacement surgery

**Fig. 4:**
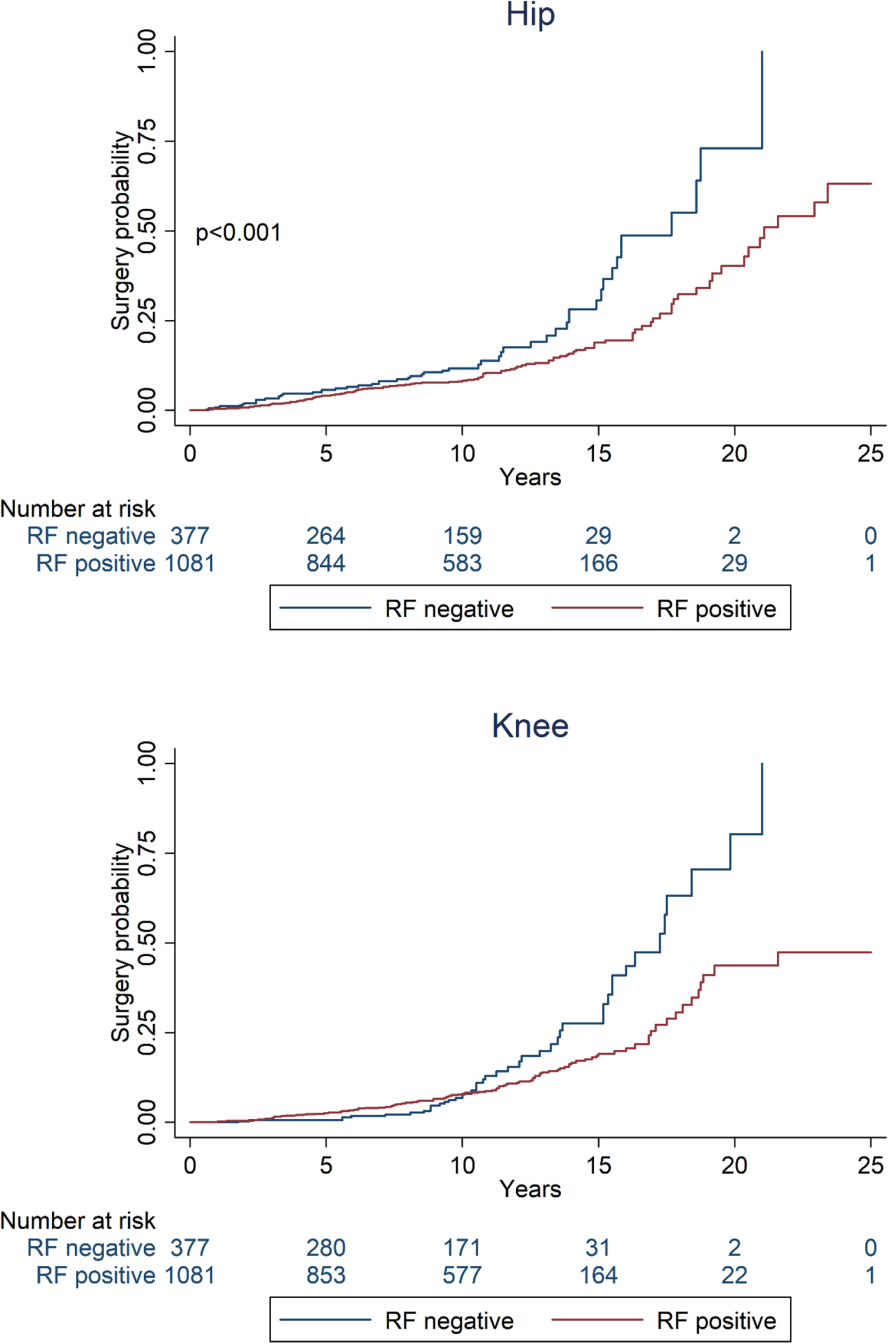
Kaplan Meier estimates of time to joint surgery at the hip and knee

The overall percentage of patients undergoing joint surgery at any site was low over 25 years’ maximum follow-up. Kaplan Meier estimates for the hip and knee are shown in Fig. 4 (other joints shown in Supplementary Figure S3). RF-positive patients had lower hazard of surgery at the elbow (HR 0.37; 95% CI 0.15, 0.90) and hip (HR 0.69; 95% CI 0.48, 0.99). For knee surgery the proportional hazards assumption was violated (curves crossed at 10 years). Cox models of years 0 to 10 showed no significant difference in hazard of knee surgery (HR 1.45; 95% CI 0.80, 2.63), whereas from year 10 onwards the hazard was HR 0.41 lower in the RF-positive participants (95% CI 0.25, 0.68). There was no significant difference in rate of surgery between RF-positive and RF-negative patients at the shoulder (HR 1.35; 0.39, 4.68), wrist (HR 0.91; 0.43, 1.92) and ankle (HR 0.86; 0.30, 2.45) (full model coefficients shown in Supplementary Table S7).

## Discussion

We present findings from ERAS, a historic inception cohort of newly diagnosed RA patients, recruited between 20 and 35 years ago, followed for a median of 10 years. This was at a time when contemporary management was very different to now; employing less intense follow-up, non-targeted treatment adjustments and less aggressive treatment choices usually not including MTX, and rarely combination csDMARD therapies. This provides the opportunity to study the natural history of this disease if under treated according to today’s standards.

We have focussed on large joint disease, which clusters separately from small joint disease [3], and has a strong influence on disability and quality of life [4, 7, 20, 21]. In ERAS we report an incremental involvement of all large joints assessed from year 3 to 14, using loss of normal ROM as a measure of RA damage. This tool is a reliable and valid measure of large joint RA with clinical utility [22], is indicative of disability [20] and correlates with standard measures of damage [21]. The prevalence of wrist disease was by far the highest, rising from 43% at year 3 to 71% of all patients at year 14; its predominance is in keeping with other cohorts [3–5, 7]. Prevalence of hip disease was the lowest, rising from 12% at year 3 to 24% at year 14, with elbow, knee and ankle much the same, rising to 26–33% of cases at year 14. There is a remarkable paucity of published information on the time course of joint involvement in RA; we believe our data from ERAS, showing the prevalence of disease, and similar odds of progression over time in all large joints is unique.

An important and perhaps surprising finding was that ankle involvement is as prevalent as other large joints, yet omitted from composite disease activity scores such as DAS28 and CDAI. Furthermore, by year 9 the ankle was the most prevalent large joint (after the wrist) to exhibit higher degrees of loss of ROM, nearly twice as prevalent as elbow and knee. A high prevalence of ankle disease in established RA has also been reported in other series, including 735 patients with established active RA used to develop the DAS28 score, where ankle involvement based on joint swelling was even higher than in ERAS, recorded in 62% compared to knee 52% and elbow 37% [3]. In another cohort of 997 patients with established RA, asked to self-report joint involvement, the prevalence of hindfoot/ankle disease was 17%, knee 32% and elbow 14%; and in early RA cohorts from four countries, prevalence of ankle involvement assessed by joint swelling ranged from 20 to 45% [5]. In an early RA cohort, elbow and ankle prevalence were both 14% and knee 25% in the sub-group with the most destructive disease at 1 year [4], not dissimilar to the ERAS prevalence at year 3 (16%). We highlight that ankle disease is prevalent in established RA, and potentially over looked if the foot is not examined, as might occur if assessment is restricted to the 28 joint count included in the DAS28 and CDAI scores. Indeed, we have reported previously that only one-third of this cohort accessed podiatry [23].

Data for RF were available for the ERAS cohort, but not ACPA as the study pre-dates its routine assessment in clinical practice. At baseline, we found a high prevalence of erosions on radiographs of wrists, MCP, PIP and MTP joints, involving 75% of the entire cohort, in keeping with other early RA cohorts [24]. Erosions were significantly less prevalent in those negative for RF (57%) compared to RF-positive (81%); also found by others [10, 24]. This might be expected from the finding that, in ERAS, there were significantly fewer ever smokers at baseline in the RF-negative versus RF-positive patients (35 vs 44%), a risk factor known to associate with erosive disease [25]. However, in contrast, other baseline poor prognostic attributes were more prevalent in RF-negative patients including a higher swollen joint count and HAQ and a higher proportion living in the most deprived areas. Despite the opposing influences of these prognostic factors, the findings in the ERAS cohort at baseline are in keeping with others, that rheumatoid destruction of the wrist and small joints is more prevalent in RF-positive patients. Similarly, the ERAS data demonstrates a significantly greater rate of progression of x-ray assessed damage and erosions at the wrist in RF-positive patients over time.

In large joints we found no significant difference in rate of loss of ROM over time between RF positive and negative cases. At year 3 we found RF-negative patients were more likely to have any loss of ROM at the hip and ankle than RF-positive patients, suggesting a more aggressive disease course at this stage. This is reflected in surgical outcomes, where RF-negative patients were found to have more procedures at the elbow and hip, and after 10 years at knee. This might be explained by a more aggressive pathology at these sites in RF-negative RA, as described in case reports [26]. Alternatively, mis-diagnosis might have influenced these outcomes, though in ERAS, patients were excluded if they later developed spondyloarthropathy or psoriatic arthritis. Furthermore, in a series of 9784 cases of seronegative RA in Finland, the diagnosis was subsequently changed to peripheral spondyloarthropathy in only 10% of patients over 15 years [27]. Osteoarthritis is known to co-exist with RA, but adjustment of the models for Lawrence assessed OA of hand and feet radiographs did not influence these results. We cannot exclude a faster time course to secondary OA in large joints of RF-negative versus positive patients. A systematic review of predictors of orthopaedic intervention in RA found no consistent influence of RF [28]. Taking these various factors into consideration, we conclude that RF-negative RA is at least as aggressive as RF-positive RA with respect to large joint involvement over time and our surgical data suggest that it may have a more destructive course, though absolute numbers of surgical interventions were low, making this a tentative observation.

The strengths of this study come from its real-world inception cohort design, with data collected from individual joints up to 14 years. Follow-up across all centres was relatively high given the long-term nature of this prospective study. Of those not followed to death or closure, cases lost to follow-up for no known reason were only 12.5%, and full reasons have been previously reported [29]. Whilst treatment strategies differ from now, our findings provide an insight into the potential disease course in large joints when treated less aggressively and provide evidence against perceptions that RF-negative RA is a less aggressive form of this disease. We would, therefore, conclude that T2T strategies employing escalating doses of MTX and combination csDMARDs should be used as much for RF-negative as RF-positive disease.

A weakness of the study is the absence of ACPA or anti-carbamylated protein antibody data, neither being routinely available through the time course of ERAS. Interestingly, quantitative high-resolution CT findings of the metacarpal head demonstrate that the association of ACPA with erosions is principally seen in RF-positive patients, whereas significantly fewer erosions were found in RF-negative cases irrespective of ACPA status [30]. Similarly, in an inception cohort followed for 6 years, IgM RF had the strongest predictive influence on progression of function (HAQ) and small joint radiologic damage whereas ACPA was only weakly predictive of damage [31]. Whether these findings also apply to large joints is unknown; the current study did not capture radiographic progression in large joints other than the wrist. The use of loss of any ROM as a reliable and valid marker of RA joint involvement [20–22] has potential limitations, as non-RA processes might incur false positive conclusions. This is particularly the case for the shoulder where rotator cuff disease is prevalent, and we, therefore, do not wish to over interpret the shoulder-specific data. This study did not assess ROM at baseline which could have shed light on progression over the first three years after diagnosis. We have previously reported in a subgroup of this cohort that patients homozygous for the HLA DR4 shared epitope (SE) are more likely to have orthopaedic surgery, although the association was not strong [32], consistent with the known relationship of the SE with severity of RA, in addition to susceptibility. In the current study, the SE was related to greater loss of ROM at 9 years, reflecting overall severity of RA, but there were only minor differences between individual large joint ROM loss and the SE. Patients with mainly large joint involvement were less likely to exhibit the SE, compared to patients with mainly polyarticular or small joint arthropathy (data not shown).

In summary, this is a unique report of the course of involvement of large joints from early to established RA, demonstrating the natural history of disease when under-treated by today’s standards. We highlight the prevalent involvement of the ankle, an under-mentioned and under-examined joint in RA, excluded from composite scores such as DAS28. We have confirmed others in demonstrating a higher burden of erosions and damage at the wrists in RF-positive patients, but not found RF-negative patients to have a better prognosis over time with respect to involvement of other large joints. In contrast, we present data to suggest that patients who are RF-negative have more joint surgery at the elbow, hip and knee after 10 years. There is no justification to adopt a less aggressive treatment strategy for RF-negative RA. High vigilance and treat-to-target approaches should be followed irrespective of RF status.

## Supplementary Information

Below is the link to the electronic supplementary material.

Supplementary file1 (DOCX 493 kb)

## Data Availability

Dataset available from Prof A. Young, Centre for Health Services and Clinical Research and Post Graduate Medicine, University of Hertfordshire, Hatfield, UK.
