# Supplementary material for: Large joints are progressively involved in rheumatoid arthritis irrespective of rheumatoid factor status—results from the early rheumatoid arthritis study
Source: Rheumatol Int. 2021 Aug 16;42(4):621–9. doi: 10.1007/s00296-021-04931-2 (PMC8940793; doi:10.1007/s00296-021-04931-2)
Supplement: Supplementary file 1 — Supplementary file1 (DOCX 493 kb) [file 296_2021_4931_MOESM1_ESM.docx]

**Supplementary materials**

For “Large joint involvement in rheumatoid arthritis progresses irrespective of rheumatoid factor status - Results from the Early Rheumatoid Arthritis Study.”

Contents

[Supplementary Figure S1: Extent of loss of range of movement for individual joints at years 3, 5, 9 and 14. 2](#_Toc74659091)

[Supplementary Table S1: Loss in range of movement in each large joint over follow-up. 5](#_Toc74659092)

[Supplementary Table S2: Full model coefficients for progression of loss of ROM over time at each joint in all participants. 6](#_Toc74659093)

[Supplementary Table S3: Full model coefficients for progression of loss of ROM over time at each joint in RF-positive participants. 7](#_Toc74659094)

[Supplementary Table S4: Full model coefficients for progression of loss of ROM over time at each joint in RF-negative participants. 8](#_Toc74659095)

[Supplementary Figure S2: Odds of progression to loss of ROM (from no loss of ROM) per year in the overall population and stratified by RF status, with models additionally adjusting for smoking (categories: never, ever, missing), rheumatic disease co-morbidity index (RDCI), and index of multiple deprivation (IMD). 9](#_Toc74659096)

[Supplementary Table S5: Annual increase in odds of developing any erosions (according to Larsen score) at the wrist. 10](#_Toc74659097)

[Supplementary Table S6: Annual change in Larsen damage score for the wrist. 11](#_Toc74659098)

[Supplementary Figure S3: Kaplan Meier estimates of time to joint surgery. 12](#_Toc74659099)

[Supplementary Table S7: Multivariable Cox models showing HR of surgical procedures to individual joints 14](#_Toc74659100)

### Supplementary Figure S1: Extent of loss of range of movement for individual joints at years 3, 5, 9 and 14.

The extent of loss of ROM was determined by the average of the left and right score for each joint region; for example, left shoulder 2, right shoulder 2 = 2 average; left ankle 0, right ankle 4 = 2 average.

The extent of loss of ROM was determined by the average of the left and right score for each joint region; for example, left shoulder 2, right shoulder 2 = 2 average; left ankle 0, right ankle 4 = 2 average.

The extent of loss of ROM was determined by the average of the left and right score for each joint region; for example, left shoulder 2, right shoulder 2 = 2 average; left ankle 0, right ankle 4 = 2 average.

### Supplementary Table S1: Loss in range of movement in each large joint over follow-up.

| **Joint** | **Any ROM loss** | **Year** | | | |
| --- | --- | --- | --- | --- | --- |
|  |  | **3** | **5** | **9** | **14*** |
| Shoulder | No | 875 (77.9%) | 693 (70.4%) | 417 (64.0%) | 101 (53.7%) |
|  | Yes | 248 (22.1%) | 292 (29.6%) | 235 (36.0%) | 87 (46.3%) |
| Elbow | No | 963 (85.1%) | 765 (77.4%) | 466 (70.9%) | 140 (74.5%) |
|  | Yes | 169 (14.9%) | 223 (22.6%) | 191 (29.1%) | 48 (25.5%) |
| Wrist | No | 634 (57.3%) | 421 (43.8%) | 244 (37.7%) | 54 (28.6%) |
|  | Yes | 472 (42.7%) | 541 (56.2%) | 404 (62.3%) | 135 (71.4%) |
| Hip | No | 996 (88.1%) | 810 (82.6%) | 494 (76.9%) | 142 (75.9%) |
|  | Yes | 134 (11.9%) | 171 (17.4%) | 148 (23.1%) | 45 (24.1%) |
| Knee | No | 960 (85.5%) | 769 (78.6%) | 456 (70.0%) | 130 (69.1%) |
|  | Yes | 163 (14.5%) | 209 (21.4%) | 195 (30.0%) | 58 (30.9%) |
| Ankle | No | 940 (84.5%) | 707 (72.6%) | 426 (65.4%) | 126 (67.0%) |
|  | Yes | 173 (15.5%) | 267 (27.4%) | 225 (34.6%) | 62 (33.0%) |
| Hind-foot | No | 937 (84.0%) | 705 (72.5%) | 424 (65.2%) | 114 (60.6%) |
|  | Yes | 179 (16.0%) | 268 (27.5%) | 226 (34.8%) | 74 (39.4%) |
| *Year 14 data came from years 12 to 15. ROM, range of movement. | | | | | |

### Supplementary Table S2: Full model coefficients for progression of loss of ROM over time at each joint in all participants.

|  | **Shoulder** | **Elbow** | **Wrist** | **Hip** | **Knee** | **Ankle** | **Hindfoot** |  |
| --- | --- | --- | --- | --- | --- | --- | --- | --- |
| No. of participants | 972 | 971 | 970 | 965 | 969 | 970 | 970 |  |
| Time (per year from year 3 to 14) | 1.10*** (1.07,1.13) | 1.07*** (1.04,1.10) | 1.09*** (1.07,1.12) | 1.10*** (1.06,1.14) | 1.12*** (1.08,1.15) | 1.11*** (1.07,1.14) | 1.13*** (1.09,1.16) |  |
| RF status^$^ (positive vs negative) | 0.81 (0.59,1.12) | 1.41 (0.97,2.05) | 1.29 (0.97,1.71) | 0.56*** (0.40,0.77) | 0.79 (0.57,1.08) | 0.58*** (0.43,0.79) | 0.78 (0.57,1.07) |  |
| Age, years | 1.02*** (1.01,1.03) | 1 (0.99,1.01) | 1.02*** (1.01,1.02) | 1.03*** (1.02,1.04) | 1.03*** (1.01,1.04) | 1.03*** (1.02,1.04) | 1.03*** (1.02,1.05) |  |
| Female (vs male) | 0.83 (0.62,1.10) | 0.50*** (0.37,0.67) | 0.88 (0.68,1.14) | 0.8 (0.59,1.09) | 0.82 (0.61,1.10) | 0.72* (0.55,0.96) | 0.95 (0.72,1.26) |  |
| BMI, kg/m2 | 1.03* (1.00,1.05) | 1.02 (1.00,1.05) | 0.98 (0.96,1.00) | 1.05*** (1.03,1.08) | 1.06*** (1.03,1.08) | 1.04*** (1.02,1.07) | 1.03** (1.01,1.06) |  |
| Baseline erosion (yes vs no) | 0.92 (0.68,1.25) | 0.96 (0.70,1.32) | 1.18 (0.89,1.55) | 0.97 (0.68,1.37) | 1.1 (0.80,1.50) | 1.01 (0.75,1.36) | 1.08 (0.80,1.46) |  |
| Erosion time varying (yes vs no) | 1.40* (1.06,1.86) | 1.92*** (1.36,2.69) | 2.15*** (1.68,2.75) | 1.34 (0.96,1.85) | 1.36* (1.01,1.82) | 1.72*** (1.25,2.38) | 1.36 (1.00,1.85) |  |
| Hb time varying | 0.97 (0.89,1.05) | 0.86*** (0.79,0.93) | 0.91** (0.85,0.98) | 1.07 (0.98,1.16) | 0.96 (0.89,1.05) | 0.95 (0.87,1.03) | 0.93 (0.85,1.01) |  |
| HAQ time varying | 2.03*** (1.74,2.36) | 1.50*** (1.28,1.76) | 1.30*** (1.13,1.49) | 1.37*** (1.15,1.63) | 1.67*** (1.41,1.98) | 1.58*** (1.35,1.85) | 1.77*** (1.51,2.08) |  |
| DAS time varying | 1.34*** (1.22,1.46) | 1.18*** (1.08,1.29) | 1.18*** (1.10,1.27) | 1.33*** (1.20,1.47) | 1.29*** (1.17,1.42) | 1.31*** (1.20,1.43) | 1.23*** (1.12,1.34) |  |
| OA hands^∆^ time varying | 0.67* (0.48,0.94) | 0.71 (0.50,1.00) | 0.85 (0.64,1.13) | 0.97 (0.70,1.35) | 1.14 (0.83,1.58) | 0.73 (0.52,1.01) | 0.78 (0.57,1.08) |  |
| OA feet^∆^ time varying | 1.25 (0.94,1.67) | 0.88 (0.65,1.20) | 1.04 (0.82,1.33) | 1.36 (0.98,1.89) | 1.02 (0.76,1.36) | 0.86 (0.64,1.16) | 0.94 (0.70,1.26) |  |
| Data shown as odds ratio (95% confidence interval). ^$^Patients were classified as RF-negative if all assessments were negative, or as RF-positive if any RF result was at least weakly positive. *** p<0.001, ** p<0.01, *p<0.05. ^∆^by Lawrence score.  DAS, disease activity score; HAQ, health assessment questionnaire; Hb, haemoglobin; OA, osteoarthritis; RF, rheumatoid factor. | | | | | | | | |

### Supplementary Table S3: Full model coefficients for progression of loss of ROM over time at each joint in RF-positive participants.

|  | **Shoulder** | **Elbow** | **Wrist** | **Hip** | **Knee** | **Ankle** | **Hindfoot** |  |
| --- | --- | --- | --- | --- | --- | --- | --- | --- |
| No. of participants | 755 | 754 | 754 | 749 | 751 | 753 | 753 |  |
| Time (per year from year 3 to 14) | 1.10*** (1.07,1.14) | 1.07*** (1.04,1.10) | 1.09*** (1.06,1.12) | 1.10*** (1.06,1.15) | 1.11*** (1.07,1.15) | 1.10*** (1.06,1.14) | 1.13*** (1.09,1.17) |  |
| Age, years | 1.02*** (1.01,1.03) | 1 (0.99,1.01) | 1.01** (1.00,1.02) | 1.03*** (1.01,1.04) | 1.02*** (1.01,1.04) | 1.03*** (1.01,1.04) | 1.03*** (1.02,1.05) |  |
| Female (vs male) | 0.89 (0.65,1.22) | 0.45*** (0.33,0.63) | 0.91 (0.68,1.21) | 0.72 (0.50,1.02) | 0.78 (0.55,1.10) | 0.84 (0.61,1.15) | 1.05 (0.76,1.44) |  |
| BMI, kg/m2 | 1.03* (1.00,1.05) | 1.02 (0.99,1.05) | 0.99 (0.97,1.01) | 1.06*** (1.03,1.09) | 1.05*** (1.03,1.08) | 1.04** (1.02,1.07) | 1.03 (1.00,1.06) |  |
| Baseline erosion (yes vs no) | 1.01 (0.73,1.39) | 1.03 (0.74,1.45) | 1.23 (0.91,1.67) | 1.17 (0.80,1.72) | 1.17 (0.83,1.64) | 1.02 (0.73,1.41) | 1.06 (0.76,1.48) |  |
| Erosion time varying (yes vs no) | 1.36 (0.98,1.89) | 1.99*** (1.32,2.99) | 2.17*** (1.65,2.86) | 1.60* (1.02,2.51) | 1.48* (1.02,2.16) | 1.56* (1.06,2.31) | 1.37 (0.95,1.98) |  |
| Hb time varying | 0.97 (0.89,1.05) | 0.84*** (0.77,0.92) | 0.89** (0.82,0.96) | 1.05 (0.95,1.16) | 0.95 (0.87,1.04) | 0.92 (0.83,1.01) | 0.92 (0.83,1.01) |  |
| HAQ time varying | 1.93*** (1.62,2.28) | 1.54*** (1.29,1.85) | 1.31*** (1.12,1.53) | 1.43*** (1.16,1.75) | 1.63*** (1.33,1.99) | 1.62*** (1.35,1.94) | 1.91*** (1.59,2.29) |  |
| DAS time varying | 1.33*** (1.21,1.47) | 1.17** (1.07,1.29) | 1.14** (1.05,1.24) | 1.24*** (1.10,1.40) | 1.27*** (1.14,1.42) | 1.27*** (1.16,1.40) | 1.19** (1.07,1.32) |  |
| OA hands^∆^ time varying | 0.72 (0.49,1.06) | 0.67 (0.45,1.00) | 0.9 (0.64,1.26) | 1.1 (0.75,1.62) | 1.44 (1.00,2.09) | 0.8 (0.54,1.17) | 0.83 (0.57,1.20) |  |
| OA feet^∆^ time varying | 1.35 (0.98,1.86) | 0.89 (0.63,1.27) | 0.97 (0.74,1.28) | 1.33 (0.91,1.94) | 0.98 (0.71,1.36) | 0.8 (0.57,1.14) | 0.96 (0.69,1.32) |  |
| Data shown as odds ratio (95% confidence interval). *** p<0.001, ** p<0.01, *p<0.05. ^∆^by Lawrence score.  DAS, disease activity score; HAQ, health assessment questionnaire; Hb, haemoglobin; OA, osteoarthritis; RF, rheumatoid factor | | | | | | | | |

### Supplementary Table S4: Full model coefficients for progression of loss of ROM over time at each joint in RF-negative participants.

|  | **Shoulder** | **Elbow** | **Wrist** | **Hip** | **Knee** | **Ankle** | **Hindfoot** |  |
| --- | --- | --- | --- | --- | --- | --- | --- | --- |
| No. of participants | 217 | 217 | 216 | 216 | 218 | 217 | 217 |  |
| Time (per year from year 3 to 14) | 1.07 (1.00,1.14) | 1.06 (0.99,1.12) | 1.11*** (1.05,1.17) | 1.07 (1.00,1.15) | 1.15*** (1.07,1.24) | 1.15*** (1.07,1.25) | 1.14*** (1.06,1.22) |  |
| Age, years | 1.02 (1.00,1.04) | 1.02 (0.99,1.05) | 1.03** (1.01,1.05) | 1.06*** (1.03,1.09) | 1.05*** (1.02,1.08) | 1.04*** (1.02,1.07) | 1.04** (1.01,1.06) |  |
| Female (vs male) | 0.66 (0.32,1.33) | 0.71 (0.35,1.44) | 0.75 (0.42,1.33) | 1.03 (0.54,1.97) | 1.04 (0.57,1.93) | 0.45** (0.25,0.81) | 0.69 (0.38,1.27) |  |
| BMI, kg/m2 | 1.03 (0.98,1.09) | 1.01 (0.95,1.06) | 0.97 (0.92,1.02) | 1.04 (0.99,1.09) | 1.08** (1.03,1.13) | 1.04 (1.00,1.09) | 1.06* (1.01,1.11) |  |
| Baseline erosion (yes vs no) | 0.57 (0.23,1.42) | 0.61 (0.25,1.49) | 1.03 (0.52,2.01) | 0.42 (0.17,1.02) | 0.88 (0.39,1.99) | 1.06 (0.55,2.07) | 1.27 (0.62,2.62) |  |
| Erosion time varying (yes vs no) | 1.68 (0.93,3.01) | 1.75 (0.96,3.17) | 2.12* (1.19,3.77) | 1.08 (0.63,1.84) | 1.09 (0.64,1.88) | 2.14* (1.19,3.84) | 1.35 (0.74,2.45) |  |
| Hb time varying | 0.97 (0.80,1.18) | 0.96 (0.80,1.16) | 0.99 (0.85,1.14) | 1.13 (0.93,1.39) | 1.01 (0.83,1.23) | 1.04 (0.88,1.22) | 0.95 (0.79,1.14) |  |
| HAQ time varying | 2.54*** (1.78,3.61) | 1.38 (0.97,1.97) | 1.22 (0.91,1.64) | 1.26 (0.91,1.74) | 2.01*** (1.44,2.80) | 1.38* (1.01,1.88) | 1.34 (0.97,1.84) |  |
| DAS time varying | 1.34** (1.10,1.62) | 1.27* (1.01,1.59) | 1.39*** (1.17,1.65) | 1.59*** (1.29,1.96) | 1.33** (1.10,1.61) | 1.50*** (1.22,1.83) | 1.40*** (1.15,1.69) |  |
| OA hands^∆^ time varying | 0.56 (0.30,1.05) | 0.91 (0.45,1.83) | 0.68 (0.39,1.18) | 0.7 (0.36,1.37) | 0.53 (0.27,1.06) | 0.53 (0.27,1.01) | 0.64 (0.33,1.25) |  |
| OA feet^∆^ time varying | 0.93 (0.52,1.65) | 0.75 (0.41,1.36) | 1.37 (0.82,2.28) | 1.46 (0.75,2.83) | 1.14 (0.58,2.22) | 1.13 (0.60,2.11) | 0.91 (0.46,1.81) |  |
| Data shown as odds ratio (95% confidence interval). *** p<0.001, ** p<0.01, *p<0.05. ^∆^by Lawrence score.  DAS, disease activity score; HAQ, health assessment questionnaire; Hb, haemoglobin; OA, osteoarthritis; RF, rheumatoid factor | | | | | | | | |

### Supplementary Figure S2: Odds of progression to loss of ROM (from no loss of ROM) per year in the overall population and stratified by RF status, with models additionally adjusting for smoking (categories: never, ever, missing), rheumatic disease co-morbidity index (RDCI), and index of multiple deprivation (IMD).


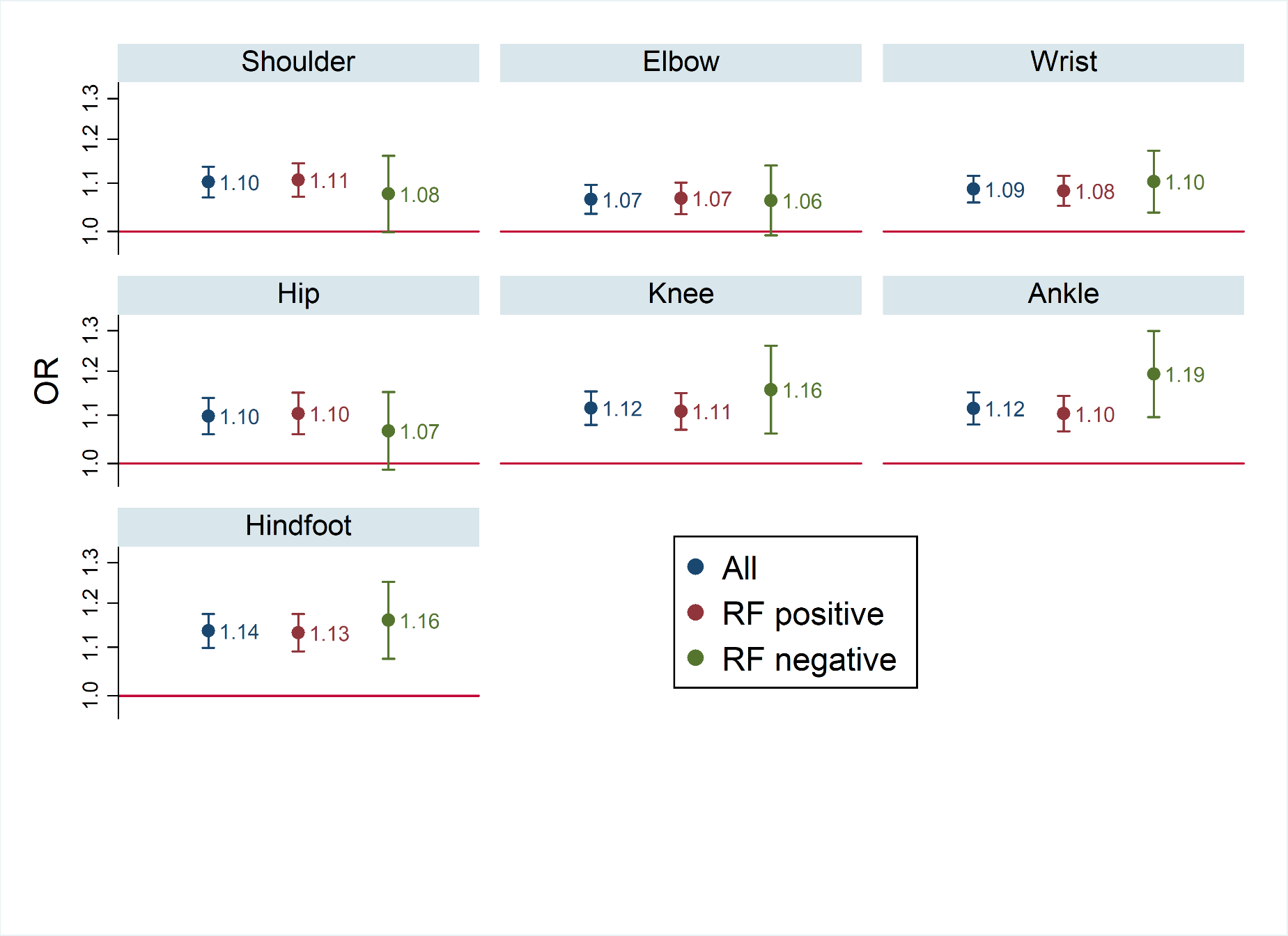


### Supplementary Table S5: Annual increase in odds of developing any erosions (according to Larsen score) at the wrist.

|  | **All patients** | **RF positive**^$^ | **RF negative** |  |
| --- | --- | --- | --- | --- |
| No. of participants | 989 | 765 | 244 |  |
| Time (per year from year 3 to 14) | 1.26*** (1.22,1.30) | 1.28*** (1.24,1.32) | 1.17*** (1.09,1.26) |  |
| RF status^$^ (positive vs negative) | 1.58* (1.11,2.25) | na | na |  |
| Age, years | 1.02*** (1.01,1.03) | 1.02*** (1.01,1.03) | 1.01 (0.98,1.03) |  |
| Female (vs male) | 1.55** (1.16,2.06) | 1.49* (1.08,2.04) | 1.94 (0.92,4.09) |  |
| BMI, kg/m2 | 0.97** (0.94,0.99) | 0.97* (0.95,1.00) | 0.92** (0.87,0.98) |  |
| Baseline erosion (yes vs no) | 1.71*** (1.29,2.27) | 1.70*** (1.25,2.31) | 1.81 (0.90,3.68) |  |
| Erosion time varying (yes vs no) | 3.92*** (3.03,5.07) | 3.73*** (2.82,4.95) | 4.75*** (2.55,8.84) |  |
| Hb time varying | 0.94* (0.88,1.00) | 0.93* (0.87,1.00) | 0.96 (0.84,1.09) |  |
| HAQ time varying | 1.25** (1.09,1.43) | 1.27** (1.09,1.48) | 1.1 (0.81,1.50) |  |
| DAS time varying | 1.02 (0.96,1.08) | 1.03 (0.96,1.10) | 0.96 (0.84,1.10) |  |
| OA hands^∆^ time varying | 0.57*** (0.43,0.77) | 0.56*** (0.40,0.78) | 0.66 (0.36,1.19) |  |
| OA feet^∆^ time varying | 0.81 (0.62,1.05) | 0.85 (0.64,1.14) | 0.63 (0.33,1.20) |  |
| Data shown as odds ratio (95% confidence interval).  ^$^Patients were classified as RF-negative if all assessments were negative, or as RF-positive if any RF result was at least weakly positive. ^∆^by Lawrence score. *** p<0.001, ** p<0.01, *p<0.05.  DAS, disease activity score; HAQ, health assessment questionnaire; Hb, haemoglobin; OA, osteoarthritis; RF, rheumatoid factor | | | | |

### Supplementary Table S6: Annual change in Larsen damage score for the wrist.

| No. of participants | 989 |
| --- | --- |
| Year | 0.63***  (0.44,0.82) |
| Rheumatoid factor^$^ (positive vs negative) | -0.12  (-0.97,0.74) |
| Year-by-RF interaction | 0.44***  (0.22,0.66) |
| Age, years | 0.07***  (0.04,0.10) |
| Female (vs male) | 1.36**  (0.46,2.27) |
| BMI, kg/m2 | -0.15***  (-0.22,-0.07) |
| Baseline erosion (yes vs no) | 3.44***  (2.11,4.78) |
| Erosion time varying (yes vs no) | 1.69***  (1.01,2.37) |
| Hb time varying | -0.07  (-0.26,0.12) |
| HAQ time varying | 0.77***  (0.32,1.21) |
| DAS time varying | 0.16  (-0.02,0.33) |
| OA hands^∆^ time varying | -1.94***  (-2.70,-1.18) |
| OA feet^∆^ time varying | -0.83*  (-1.61,-0.05) |
| Data shown as beta coefficients (95% confidence interval), indicating change in Larsen damage score.  ^$^Patients were classified as RF-negative if all assessments were negative, or as RF-positive if any RF result was at least weakly positive. ^∆^by Lawrence score. *** p<0.001, ** p<0.01, *p<0.05.  DAS, disease activity score; HAQ, health assessment questionnaire; Hb, haemoglobin; OA, osteoarthritis; RF, rheumatoid factor | |

### Supplementary Figure S3: Kaplan Meier estimates of time to joint surgery.


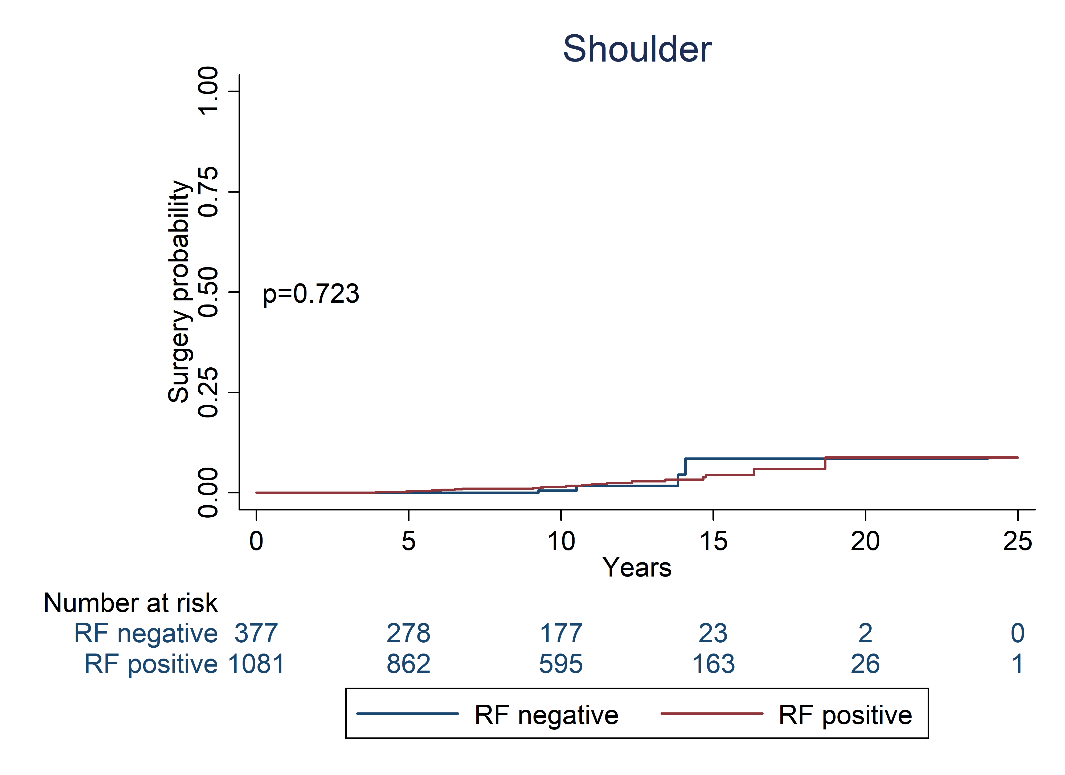

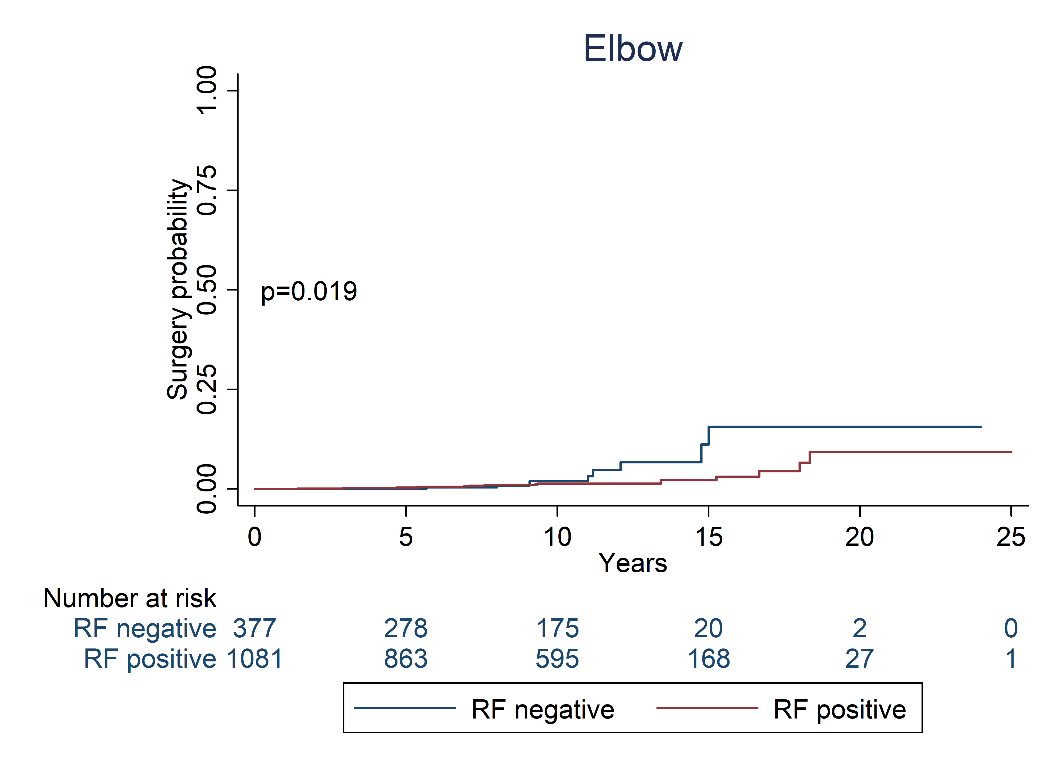


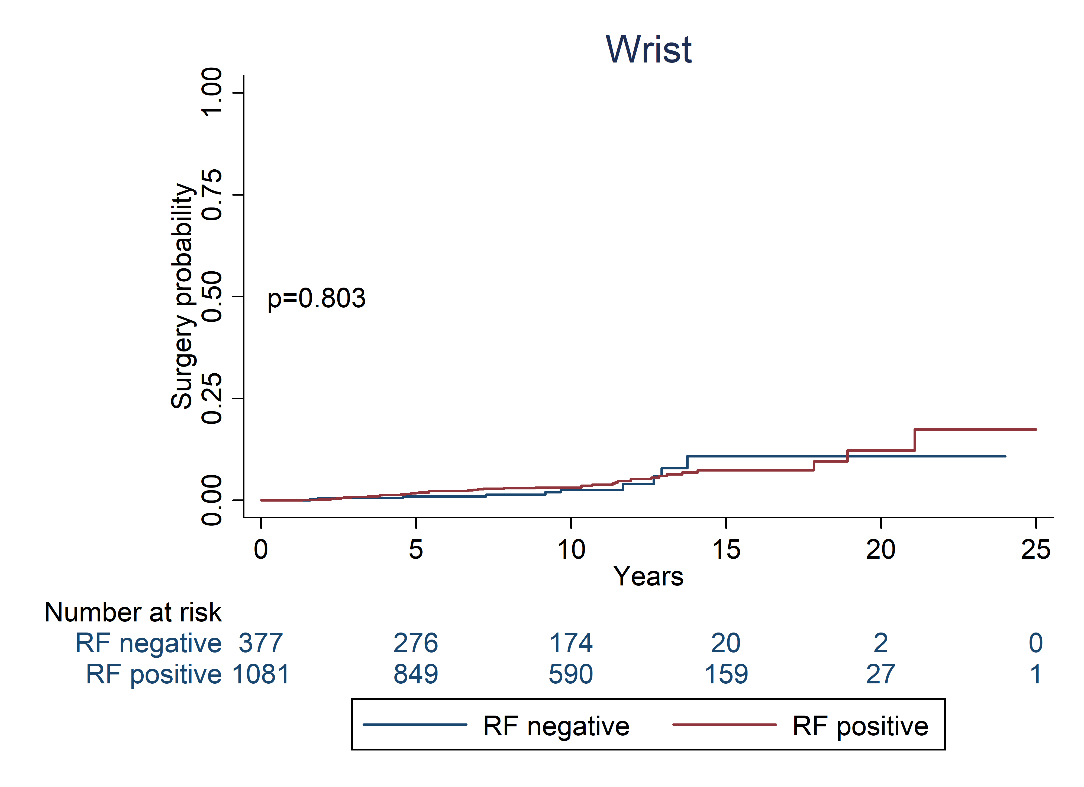

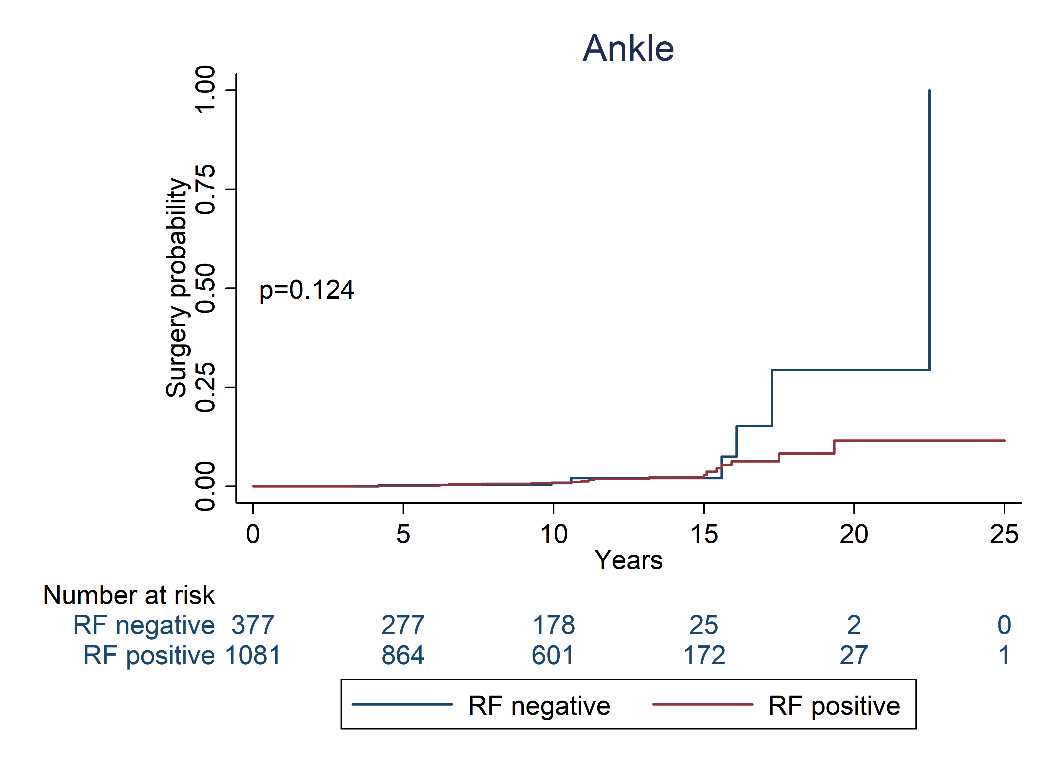


### Supplementary Table S7: Multivariable Cox models showing HR of surgical procedures to individual joints

|  | | **Shoulder** | **Elbow** | **Wrist** | **Hip** | **Ankle** | **Knee<10** | **Knee>=10** |
| --- | --- | --- | --- | --- | --- | --- | --- | --- |
| No. of participants | | 1267 | 1267 | 1267 | 1267 | 1267 | 1267 | 451 |
| RF^$^ (positive vs neg) | | 1.35 (0.39,4.68) | 0.37* (0.15,0.90) | 0.91 (0.43,1.92) | 0.69* (0.48,0.99) | 0.86 (0.30,2.45) | 1.45 (0.80,2.63) | 0.41*** (0.25,0.68) |
| Age, years | | 1.00 (0.96,1.03) | 0.99 (0.96,1.02) | 0.99 (0.97,1.02) | 1.05*** (1.03,1.06) | 0.97 (0.94,1.00) | 1.04*** (1.02,1.06) | 1.02 (1.00,1.04) |
| Female (vs male) | | 1.21 (0.46,3.16) | strat | 2.72* (1.20,6.16) | 1.31 (0.90,1.90) | 1.29 (0.42,3.95) | 1.61 (0.92,2.81) | 1.16 (0.68,1.98) |
| BMI, kg/m2 | | 0.99 (0.90,1.10) | 0.96 (0.86,1.06) | 0.94 (0.87,1.01) | 0.98 (0.95,1.02) | 1.01 (0.92,1.12) | 1.06* (1.01,1.12) | 1.08** (1.03,1.13) |
| Smoking status | Never | ref | ref | ref | ref | ref | ref | ref |
|  | Ever | strat | 1.16 (0.45,2.99) | 1.66 (0.85,3.25) | strat | 0.44 (0.12,1.60) | 0.86 (0.48,1.54) | 1.15 (0.65,2.04) |
|  | Missing | strat | 1.49 (0.49,4.50) | 1.74 (0.79,3.83) | strat | 3.78* (1.36,10.45) | 1.37 (0.77,2.44) | 2.92*** (1.59,5.35) |
| strat – variable stratified due to PH violation.  ^$^Patients were classified as RF-negative if all assessments were negative, or as RF-positive if any RF result was at least weakly positive. *** p<0.001, ** p<0.01, *p<0.05. RF, rheumatoid factor. | | | | | | | | |
